# Characterization and Antioxidant Activity of a Low-Molecular-Weight Xanthan Gum

**DOI:** 10.3390/biom9110730

**Published:** 2019-11-12

**Authors:** Xiaolong Hu, Kangli Wang, Miao Yu, Peixin He, Hanzhen Qiao, Huiru Zhang, Zichao Wang

**Affiliations:** 1 College of Food and Biological Engineering, Zhengzhou University of Light Industry, Zhengzhou 450002, China; 2 College of Biological Engineering, Henan University of Technology, Zhengzhou 450001, China

**Keywords:** Xanthan gum, FT-IR and NMR analysis, X-ray diffraction (XRD) analysis, Caco-2 cells

## Abstract

In the present work, a low-molecular-weight xanthan gum (LW-XG) was successfully obtained via biodegradation of commercial xanthan by the endophytic fungus *Chaetomium* *globosum* CGMCC 6882. The monosaccharide composition of LW-XG was glucose, mannose, and glucuronic acid in a molar ratio of 1.63:1.5:1.0. The molecular weight of LW-XG was 4.07 × 10^4^ Da and much smaller than that of commercial xanthan (2.95 × 10^6^ Da). Antioxidant assays showed that LW-XG had a good scavenging ability on DPPH radicals, superoxide anions, and hydroxyl radicals and good ferric reducing power. Moreover, LW-XG exhibited excellent protective effect on H_2_O_2_-injured Caco-2 cells. Results of this work suggested that LW-XG could be used in foods or pharmaceuticals to alleviate and resist the oxidative damage induced by the overproduction of reactive oxygen species.

## 1. Introduction

Xanthan gum, which is produced from aerobic fermentation by *Xanthomonas campestris*, is composed of a β-1,4-glycosidic bond-linked main chain and a trisaccharide side chain successively containing mannose, glucuronic acid, and mannose. Approximately, half of the terminal mannose contains a pyruvic acid residue with unknown distribution [1]. Due to its high molecular weight (2.0 × 10^6^–2.0 × 10^7^ Da) and unique chemical structure, xanthan gum shows excellent pseudoplasticity, thickening, and rheological properties, and is highly stable to heat, acid, and alkali [2]. Therefore, xanthan gum is widely used as a thickener, rheological modifier, stabilizer, and emulsifier in food, textile, cosmetic, pharmaceutical, and oil recovery industries [3].

Recently, there has been a growing interest in tailoring polysaccharides for different applications, among them, reducing or confining the molecular weight of xanthan gum within a narrow range has attracted much attention. For instance, Han et al. [4] reported that low-molecular-weight xanthan gum could be used for treating osteoarthritis. Chen et al. [5] found that low-molecular-weight xanthan gum could suppress oxidative stress-induced cell apoptosis. Lower range molecular weight xanthan gum could inhibit cartilage matrix destruction and apoptosis of chondrocytes [6,7]. Meanwhile, xanthan oligosaccharides prepared by hydrolysis of commercial xanthan with hydrogen peroxide in an alkaline solution showed a high hydroxyl radical scavenging activity [8]. Xiong et al. [9] demonstrated that water-soluble xanthan oligosaccharides prepared by oxidative degradation of commercial xanthan under acidic and alkaline conditions exhibited good antioxidant activity. Moreover, Hashemi et al. [10] suggested that lysozyme–xanthan gum conjugates could not only inhibit the growth of *Staphylococcus aureus* and *Escherichia coli*, but also showed excellent antioxidant properties.

Compared to the production of toxic compounds, severe corrosion to equipment, high energy consumption, and high requirements for instruments by using physical and/or chemical methods for lower molecular weight xanthan gum preparation [11,12], biodegradation has the advantages of mild reaction conditions, less pollution, and less investment. Therefore, in the present work, the endophytic fungus *Chaetomium globosum* CGMCC 6882 was used to biodegrade commercial xanthan, and a biodegradation product was obtained after extraction and purification. Then, monosaccharide composition, molecular weight, functional groups, and chemical bonds of this biodegradation product were analyzed for confirming that it is xanthan gum, but with a low molecular weight. Finally, antioxidant activities of LW-XG were evaluated in vitro and on the H_2_O_2_-injured Caco-2 cells.

## 2. Materials and Methods

### 2.1. Strain and Materials

*C. globosum* CGMCC 6882 was isolated from the *Gynostemma pentaphyllum* herb and stored in the China General Microbiological Culture Collection Center. Chemicals were analytical grade and bought from Sinopharm Chemical Reagent Co., Ltd. (Beijing, China). Commercial xanthan gum was bought from Zibo Zhongxuan Biochemistry Co. Ltd. (Shandong, China). Cellulose Congo red medium was bought from Qingdao Hope Bio-Technology Co., Ltd. (Shandong, China).

### 2.2. Biodegradation of Commercial Xanthan

Seed medium for *C. globosum* CGMCC 6882 was prepared as follows: One square centimeter of a PDA plate with mycelia was chipped off and transferred into a 250 mL Erlenmeyer flask with 50 mL medium (20 g/L glucose, 1 g/L peptone, 1 g/L beef extract, and 1 g/L yeast extract, pH = 7.0 ± 0.2); shaking speed was 150 r/min, culture temperature was 28 °C, and culture time was 36 h.

Fermentation medium contained (g/L) commercial xanthan gum 10, (NH_4_)_2_SO_4_ 1.0, KH_2_PO_4_ 1.5, K_2_HPO_4_ 2.0, NaCl 0.5, CaCl_2_ 0.5, MgSO_4_ 1.0, FeSO_4_ 0.01, MnSO_4_ 0.01, ZnSO_4_ 0.01, CoCl_2_ 0.01, and peptone 1.0. Fermentation temperature was 28 °C, inoculation volume was 1% (v/v), cultivation time was 6 days, and pH was maintained at 7.0 ± 0.02 with 2 mol/L NaOH and 2 mol/L HCl. A 7 L fermentor (LiFlus GM BioTRON, Korea) was used. It contained 4 L medium, aeration rate was 1.0 vvm, and stirring speed was 150 r/min.

### 2.3. Extraction and Purification of LW-XG

At the end of fermentation, culture broth was added to alcohol to a concentration of 75% (v/v), then centrifuged at 10,000× *g* for 30 min and supernatant was collected. After that, the supernatant was added to alcohol to a concentration of 90% (v/v) and kept at 4 °C overnight to precipitate crude LW-XG. Crude LW-XG was redissolved in 100 mL of distilled water and deproteinized by adding three volumes of Sevag solution. Then, nine volumes of cold alcohol were added into the deproteinized supernatant and kept at 4 °C overnight to precipitate LW-XG. The precipitated LW-XG was washed three times with 90% cold alcohol and lyophilized. After that, the lyophilized LW-XG was redissolved in distilled water and depigmented with the AB-8 macroporous resin. The obtained LW-XG solution was dialyzed for 48 h in distilled water (M_W_ cut-off was 15 kDa). Finally, LW-XG solution was filtered through a 0.22 μm filter and applied to a Sepharose CL-6B column (2.5 × 60 cm) for further purification. This process was monitored by the phenol-sulfuric acid method at 490 nm absorbance, and the fraction was collected and lyophilized.

### 2.4. Monosaccharide Composition Analysis

The monosaccharide composition was analyzed using high-performance anion-exchange chromatography (HPAEC). Briefly, xanthan samples were dissolved in trifluoroacetic acid (TFA, 2 mol/L) and hydrolyzed at 120 °C for 2 h. The hydrolysate was washed three times with methanol and evaporated. Finally, the hydrolyzed material was transferred to a 25 mL volumetric flask, diluted to 25 mL by deionized water, and subjected to a Dionex ICS5000 system (Dionex, Sunnyvale, CA, USA) equipped with CarboPac PA20 column (ID 3 mm × 150 mm) [13]. The pyruvate group and acetyl group in the xanthan samples were detected by the 2,4-dinitrophenylhydrazone method and hydroxamic acid method, respectively [14].

### 2.5. FT-IR and NMR Analysis

A Nexus 470 FT-IR spectrophotometer (Nicolet, Waltham, MA, USA) was used to record the IR spectra of xanthan samples with KBr between 400–4000 cm^−1^, and a Bruker Avance 600 MHz spectrometer (Bruker Inc., Karlsruhe, Germany) was used to record the ^1^H NMR and ^13^C NMR spectra of xanthan samples at 30 °C. The chemical shifts for ^1^H NMR and ^13^C NMR spectra were recorded in parts per million by using tetramethylsilane as the internal standard [15].

### 2.6. X-ray Diffraction (XRD) Analysis

An X-ray diffractometer (D8advance, Bruker, Germany) was used to further analyze the structure of xanthan samples. The source of radiation was Cu-*K*α, scanning angle range was 10–60° (2θ), scanning voltage was 30 kV, scanning current was 30 mA, scanning rate was 2°/min, and step size was 0.02°.

### 2.7. Molecular Weight Analysis

Xanthan samples were dissolved in distilled water to a concentration of 2 mg/mL and analyzed using high-performance size exclusion chromatography (HPSEC). HPSEC consisted of the Waters 2695 HPLC system equipped with a refractive index detector (RI), a UV detector, and a multiple angle laser light scattering detector (MALLS, DAWNHELEOS, Wyatt Technology, Santa Barbara, CA, USA) [16].

### 2.8. Rheological Analysis

Xanthan samples were dissolved in 1 g/L NaCl to a concentration of 1.0% (w/v) and homogenized with magnetic stirring at room temperature overnight, then centrifuged at 10,000× *g* for 30 min to remove bubbles in the solution. Rheological properties of xanthan samples were detected by a DHR-1 dynamic shear rheometer (TA, Milford, MA, USA) at 25 °C. Steady shear viscosity was detected over a shear rate range of 0.01–100 1/s and rheological data of xanthan solutions were calculated according to the fluid power-law equation of Ostwald–de Waele model: *μ* = *Kγ*^(n−1)^ [1], where *μ* is the shear viscosity, *K* is the consistency index, *γ* is the shear rate, and n is the behavior index.

### 2.9. Antioxidant Activity Assay In Vitro

LW-XG was dissolved in distilled water to concentrations of 0.5, 1.0, 1.5, 2.0, 2.5, and 3.0 mg/mL. The antioxidant activities of LW-XG *in vitro* were analyzed by scavenging DPPH radicals, superoxide anions, and hydroxyl radicals and detecting ferric reducing power. Meanwhile, ascorbic acid (Vc) was used as a positive control in these experiments.

#### 2.9.1. DPPH Radical Scavenging Activity Assay

The DPPH radical scavenging activity of LW-XG was detected according to the method reported by Tang et al. [17] with some modifications. DPPH was dissolved in alcohol to a concentration of 0.1 mmol/L and 2 mL of alcoholic DPPH was added to 2 mL of LW-XG solution. The system was fully mixed and kept in dark for 30 min at room temperature. Finally, absorbance of the mixture was measured at 517 nm with a microplate reader (Thermo, Waltham, MA, USA). DPPH radical scavenging activity (%) = (A_0_ − A_i_ + A_j_)/A_0_ × 100%, where A_0_ is the absorbance of blank (LW-XG was replaced by alcohol), A_i_ is the absorbance of the LW-XG, and A_j_ is absorbance of the background (DPPH was replaced by alcohol).

#### 2.9.2. Superoxide Anion Scavenging Activity Assay

The superoxide anion scavenging activity of LW-XG was detected according to the method reported by Chen et al. [18] with some modifications. Tris-HCl buffer (2.5 mL, 0.05 mol/L, pH = 8.2) was added to 0.4 mL LW-XG solution. The mixture solution was reacted at 25 °C for 10 min, then 0.1 mL pyrogallic acid was added into the system and reacted for another 20 min. The reaction was quenched by adding 0.5 mL HCl and absorbance of the mixture was measured at 380 nm. Superoxide anion scavenging activity (%) = (1 − A/A_0_) × 100%, where A is the absorbance of LW-XG and A_0_ is the absorbance of blank (LW-XG was replaced by distilled water).

#### 2.9.3. Hydroxyl Radical Scavenging Activity Assay

The hydroxyl radical scavenging activity of LW-XG was detected according to the method reported by Tang et al. [19] with some modifications. Briefly, 2 mL of FeSO_4_ (6 mmol/L) and 2 mL of H_2_O_2_ (6 mmol/L) were added to 2 mL of LW-XG solution, then the system was fully mixed and reacted at room temperature for 10 min. After which, 2 mL of ortho-hydroxybenzoic acid (6 mmol/L) was added to the system and reacted for another 30 min. The absorbance of the mixture was measured at 510 nm. Hydroxyl radical scavenging activity (%) = (A_0_ − A_i_ + A_j_)/A_0_ × 100%, where A_0_ is the absorbance of LW-XG replaced by distilled water, A_i_ is the absorbance of LW-XG, and A_j_ is the absorbance of H_2_O_2_ group replaced by distilled water.

#### 2.9.4. Reducing Power Assay

The reducing power of LW-XG was detected according to the method reported by Chen et al. [20] with some modifications. Firstly, 2.5 mL of phosphate buffer (0.2 mol/L, pH = 6.6) and 2.5 mL of 1% (w/v) potassium ferricyanide were added to 2 mL of LW-XG solution, the system was mixed, and reacted at 50 °C for 20 min. At the end of the reaction, 2.5 mL of 10% (w/v) trichloroacetic acid was added to the mixture and centrifuged at 3000 r/min for 10 min. After centrifugation, 3 mL of the supernatant was pipetted and mixed with 3 mL of distilled water and 0.5 mL of 0.1% (w/v) ferric chloride. This mixture was fully mixed and reacted at room temperature for another 10 min, the mixture was then centrifuged at 5000 r/min for 10 min, and the absorbance of the supernatant was detected at 700 nm.

### 2.10. Antioxidant Activity on H_2_O_2_-Induced Injury in Caco-2 Cells

#### 2.10.1. Cytotoxicity of LW-XG on Caco-2 Cells

The cytotoxicity of LW-XG on Caco-2 cells was measured according to the method reported in our previous work [21]. Briefly, Caco-2 cells were seeded onto 96-well plates with a density of 5 × 10^4^ cells/mL and incubated at 37 °C and 5% CO_2_ for 24 h. Then, 100 μL of LW-XG solution (0.125, 0.25, 0.5, 1.0, 2.0, and 4.0 mg/mL) was added into the wells for another 24 h. The cytotoxicity result was expressed as cell viability which was determined by Cell Counting Kit-8 (CA1210-500T, Solarbio, Beijing, China).

#### 2.10.2. Injured Cell Model Induced by H_2_O_2_

In order to study the protective effect of LW-XG on oxidative damage on cells, Caco-2 cells were seeded onto 96-well plates with a density of 5 × 10^4^ cells/mL and incubated at 37 °C with 5% CO_2_. After an incubation time of 24 h, cell medium was pipetted off and new medium containing different concentrations of H_2_O_2_ (100, 200, 300, 400, 500, 600, 700, and 800 μmol/L) was added. After H_2_O_2_ treatment for 4 h, the concentration of H_2_O_2_ at which approximately 50% of the treated cells remained viable compared to a control group (without H_2_O_2_ addition) was considered to be the appropriate H_2_O_2_ concentration for establishing an injured Caco-2 cell model. The cell viability was detected by Cell Counting Kit-8.

#### 2.10.3. Effects of LW-XG on SOD, CAT, GSH-Px and MDA Activity

Caco-2 cells with a density of 1 × 10^5^ cells/mL were seeded onto 12-well plates and incubated at 37 °C with 5% CO_2_ for 24 h. The medium was abandoned and new medium containing different concentrations of LW-XG (0.75, 1.5, and 3.0 mg/mL) was added and incubated for another 24 h. Then, the medium was removed and new medium containing an appropriate concentration of H_2_O_2_ was added and incubated for 4 h. After 4 h of incubation, the medium in the 12-well plates was discarded and the cells were washed twice with cold PBS for collecting cells. The collected Caco-2 cells were lysed by a lysis buffer for catalase (CAT), superoxide dismutase (SOD), glutathione peroxidase (GSH-Px), and malondialdehyde (MDA) activity determination according to the instruction in kits. The commercial kits for detecting CAT, SOD, MDA, and GSH-Px were bought from Jiancheng Biochemical Reagent Co. Ltd. (Nanjing, China). The concentration of protein was measured by using the bicinchoninic acid protein assay kit (Beyotime Institute of Biotechnology, Shanghai, China), and the absorbance was determined with a microplate reader (Thermo, Waltham, MA, USA).

### 2.11. Statistical Analysis

All data were expressed as mean ± SD after three repeats. Data were subjected to one-way analysis of variance (ANOVA) with Duncan’s multiple range tests. A *P*-value < 0.05 was considered to be significant, following SPSS software analysis.

## 3. Results and Discussion

### 3.1. Production of LW-XG

**Figure 1:**
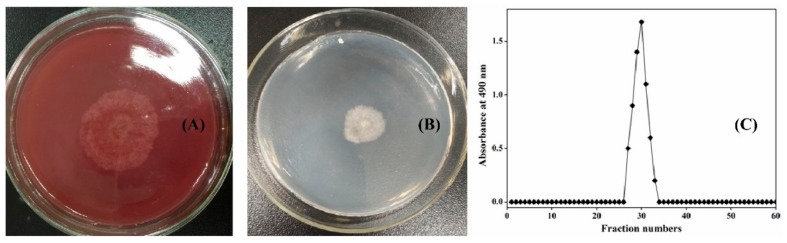
Growth profiles of *Chaetomium globosum* CGMCC 6882 on cellulose Congo red medium plate (**A**) and xanthan gum plate (**B**). Elution profile of biodegradation product by Sepharose CL-6B chromatography (**C**).

As the third common fungus after *Penicillium* and *Aspergillus* [22], *Chaetomium globosum* is widely distributed in the natural world and has the ability to secrete cellulases for cellulose degradation [23]. This was verified by the growth profile of *C. globosum* CGMCC 6882 on a cellulose Congo red medium plate (Figure 1A). Interestingly, *C. globosum* CGMCC 6882 could also use xanthan gum as the sole carbon source for growth (Figure 1B). After biodegradation, the culture supernatant was subjected to gradient alcohol sink, centrifugation, deproteinization, depigmentation, and finally, purification by a Sepharose CL-6B column. The biodegradation product had a characteristic absorption at 490 nm (Figure 1C).

### 3.2. Monosaccharide Composition

**Figure 2:**
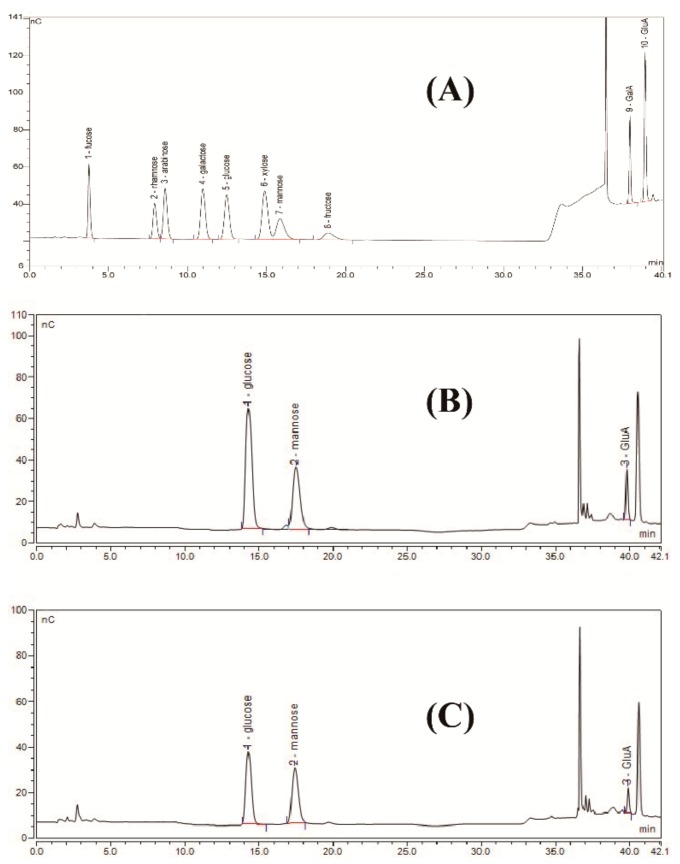
High-performance anion-exchange chromatograms of standard monosaccharides (**A**), commercial xanthan (**B**), and biodegradation product (**C**).

Compared to the standard monosaccharides used in this work (Figure 2A), HPAEC results showed that the biodegradation product was only composed of glucose, mannose, and glucuronic acid with a molar ratio of 1.63:1.5:1.0 (Figure 2C). This monosaccharide composition was the same as commercial xanthan (Figure 2B), and its molar ratio was similar to the molar ratio of commercial xanthan (1.93:1.58:1.0) and the theoretic molar ratio of xanthan gum (2.0:2.0:1.0). Furthermore, acetyl group and pyruvate group contents in the biodegradation product were 4.35% and 4.22%, respectively. These results were similar to the contents of acetyl group (4.28%) and pyruvate group (4.46%) in commercial xanthan and the results reported by Kang et al. [14]. For xanthan production, the monosaccharide composition and the molar ratio of xanthan gum were affected by many factors, such as carbon sources, strains, fermentation conditions, and extraction conditions [24,25]. In contrast, for xanthan gum degradation, irrespective of the method chosen for enzymatic, chemical, and/or physical degradation [11,12], no other monosaccharides except glucose, mannose, and glucuronic acid will be found. In the present work, the monosaccharide composition of the biodegradation product was coincident with the typical monomers of xanthan gum, preliminarily indicating that the biodegradation product is xanthan gum.

### 3.3. FT-IR

**Figure 3:**
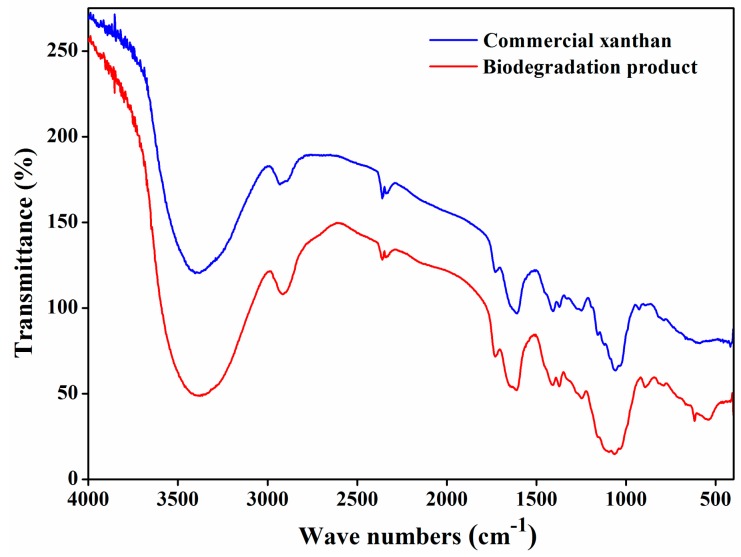
FT-IR spectra of commercial xanthan and biodegradation product.

The chemical structure of the biodegradation product was analyzed by FT-IR between 400 cm^−1^ and 4000 cm^−1^. As shown in Figure 3, broad absorption at around 3400 cm^−1^ might be due to O–H stretching, and the absorption band at 2900 cm^−1^ might refer to C-H stretching. The absorption band at around 1700 cm^−1^ might relate to carboxyl in xanthan gum and the absorption band at around 1600 cm^−1^ might be due to C=O stretching in the pyruvate group of xanthan gum. Peaks at around 1500 cm^−1^, 1400 cm^−1^, and 1100 cm^−1^ might correspond to C=C stretching, C–H stretching, and C–O–C stretching, respectively. The absorption bands between 500 cm^−1^ and 1000 cm^−1^ could be due to the stretching vibrations of =C–H, C–O, O–H, and C–C bonds [13,14,26]. The infrared spectra of the biodegradation product were consistent with those of commercial xanthan, further suggesting that the biodegradation product is xanthan gum.

### 3.4. NMR Analysis

**Figure 4:**
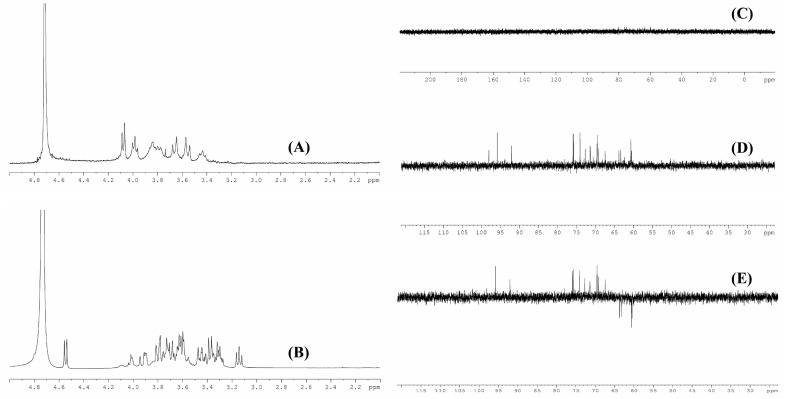
^1^H NMR (**A**) and ^13^C NMR (**C**) spectra of commercial xanthan (**A**); ^1^H NMR (**B**), ^13^C NMR (**D**), and dept 135 ^13^C NMR (**E**) spectra of biodegradation product.

The chemical bonds of the biodegradation product were further analyzed by NMR. As shown in Figure 4A,B, chemical shifts at around 4.79 ppm could be due to D_2_O used in the work, peaks between 3.8 ppm and 4.1 ppm might be attributed to the hydroxyl group in xanthan gum. The peaks at around 3.6 ppm and 3.4 ppm might correspond to CH_2_ in glucuronic acid [13]. The absorption peaks of acetate group and pyruvate group might appear at around 1.2 ppm and 2.0 ppm [14], but these two peaks were not found in the present work; this might be due to the internal standard tetramethylsilane used in this work. Due to its high molecular weight and high viscosity, ^13^C NMR analysis of commercial xanthan did not yield any peaks (Figure 4C). For the biodegradation product (Figure 4D), the peaks at around 100, 76, 73, 70, and 62 ppm might relate to C1, C3, C2, C4, and C6, respectively [27]. From the dept 135 ^13^C NMR spectra of the biodegradation product (Figure 4E), peaks at around 61 ppm and 63 ppm might relate to CH_2_, and these singles in the opposite amplitude were possibly corresponding to CH in this biodegradation product [28,29]. The results of monosaccharide composition and FT-IR analyses indicated that the biodegradation product could almost be confirmed as xanthan gum.

### 3.5. XRD Analysis

**Figure 5:**
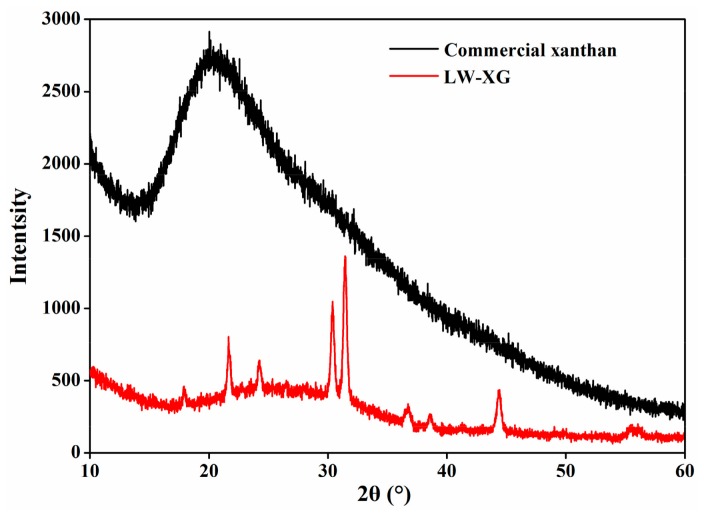
XRD patterns of commercial xanthan and LW-XG.

The XRD patterns of commercial xanthan and LW-XG are shown in Figure 5. There were no sharp peaks found when 2θ was about 20° for commercial xanthan, indicating its amorphous pattern and this is in accordance with the previous amorphous nature of xanthan gum reported by other researchers [14,30]. In contrast, the XRD spectra for LW-XG showed several clear sharp peaks at 2θ = 23°, 25°, 31°, 32°, 37.5°, and 45°, suggesting the semi-crystallographic pattern of LW-XG [30,31].

### 3.6. Molecular Weight and Rheological Analysis

**Figure 6:**
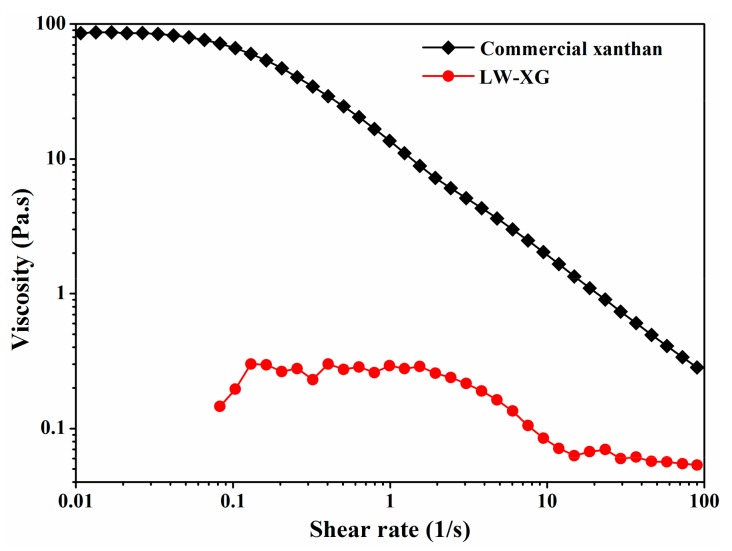
Viscosity of commercial xanthan and LW-XG at 1.0% (w/v) concentration.

HPSEC results showed that the molecular weight of LW-XG was 4.07 × 10^4^ Da, which is almost 1/100th of the weight of commercial xanthan (2.95 × 10^6^ Da). The decrease in molecular weight suggests that LW-XG might have more active groups exposed and more biological activity [8,9]. Meanwhile, the consistency index of commercial xanthan was 19.9375, but the consistency index of LW-XG could not be detected, and no viscosity could be detected at a low shear rate (Figure 6). Xanthan gum is used as a thickener in oil and paint industries because of its high thickening property at low concentrations [2]. However, low or no viscosity of LW-XG could increase and facilitate its usage in food, agriculture, pharmaceutical, and other fields.

### 3.7. Antioxidant Activity Assay In Vitro

**Figure 7:**
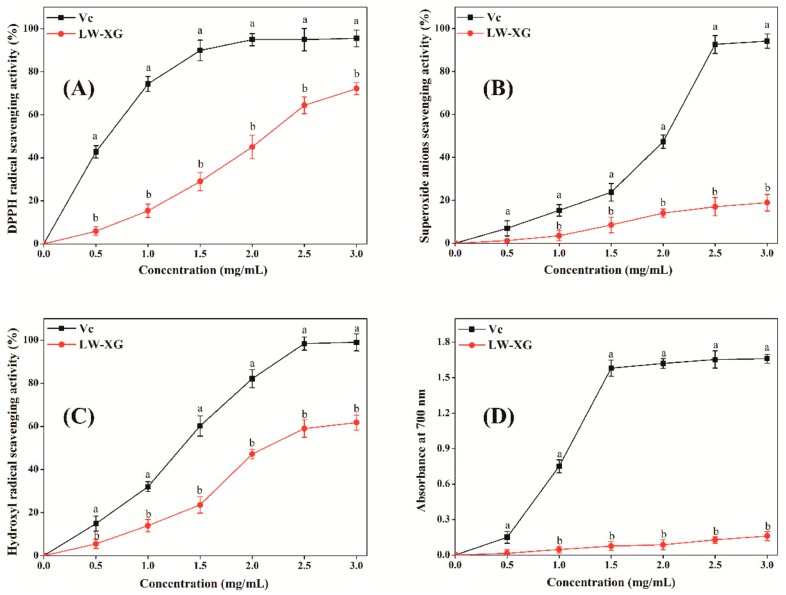
Scavenging effects of LW-XG on DPPH radicals (**A**), superoxide anions (**B**), hydroxyl radicals (**C**), and ferric reducing power (**D**). Different letters represent a significant difference at *P* < 0.05.

Oxidative stress induced by the overproduction of reactive oxygen species will damage biomolecules of DNA, proteins, and lipids, thus leading to the increased risk of cancer, cardiovascular diseases, cell apoptosis, and other diseases [32]. As can be seen from Figure 7, LW-XG showed good scavenging activity on DPPH radicals, superoxide anions, and hydroxyl radicals and good ferric reducing power; its antioxidant activity increased in a concentration-dependent manner. When the concentration of LW-XG was 3.0 mg/mL, the DPPH radical scavenging activity, superoxide anion scavenging activity, and hydroxyl radical scavenging activity were 72.2 ± 2.81%, 18.9 ± 3.05%, and 61.8 ± 2.98%, respectively, and ferric reducing power at the absorption of 700 nm was 0.2 ± 0.04. These results were similar to that observed for polysaccharides extracted from the rhizome of *Dryopteris crassirhizoma* Nakai by Zhao et al. [33], and were competitive with the antioxidant activities of xanthan oligosaccharides reported by other researchers [8,9]. The excellent antioxidant activity of LW-XG could be interpreted by the increase in hydroxyl and carboxyl groups with the decrease in molecular weight, wherein the hydroxyl and carboxyl groups combine with radicals to form stable radicals to terminate the radical chain reaction [34,35].

### 3.8. Effect of LW-XG on H_2_O_2_-Induced Injury in Caco-2 Cell Model

**Figure 8:**
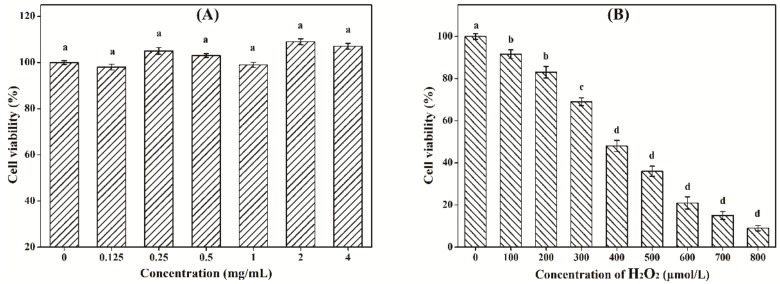
Cytotoxicity of LW-XG on Caco-2 cells (**A**) and test of H_2_O_2_ concentration on injured Caco-2 cells model (**B**). Different letters represent a significant difference at *P* < 0.05.

As shown in Figure 8A, under the tested concentrations, LW-XG exhibited no obvious toxic effect on Caco-2 cells. This result could be explained by the safety of xanthan gum approved by FDA [12]. Meanwhile, polysaccharides extracted from *Ganoderma lucidum* had no toxic effect on Caco-2 cells [21]; low-molecular-weight procyanidins extracted from grape seeds not only showed no toxic effect on Caco-2 cells, but also enhanced the impact of 5-fluorouracil chemotherapy [36]. Furthermore, the beneficial effects of the probiotic *Bacillus subtilis* on Caco-2 cells had also been verified [37,38]. These results were similar to that observed for LW-XG in this work. Figure 8B showed that the cell viability of Caco-2 cells decreased with increasing concentrations of H_2_O_2_. When H_2_O_2_ concentrations were 100 μmol/L and 200 μmol/L, cell viabilities were 91.6 ± 2.13% and 83.7 ± 2.81%, respectively, which indicated that low concentrations of H_2_O_2_ did not reduce cell viability and hence, could not be used to produce an injured Caco-2 cell model. When H_2_O_2_ concentration was increased to 400 μmol/L, the cell viability decreased to 48.4 ± 2.75%, this concentration was appropriate and could be selected for the establishment of the H_2_O_2_-injured Caco-2 cell model [32].

Due to deficiencies in the accuracy and authenticity of chemiluminescence methods, cell models are increasingly used by researchers to analyze the antioxidant activity of polysaccharides. As shown in Table 1, after the Caco-2 cells were incubated with LW-XG (0.75, 1.5, and 3.0 mg/mL) for 24 h and injured by 400 μmol/L of H_2_O_2_ for 4 h, the levels of SOD, CAT, GSH-Px, and MDA in Caco-2 cells of the LW-XG group increased in a concentration-dependent manner. When Caco-2 cells were pretreated with 3.0 mg/mL of LW-XG for 24 h and then injured by 400 μmol/L H_2_O_2_ for another 4 h, the levels of SOD, CAT, GSH-Px and MDA of the LW-XG group recovered to 51.4 ± 2.63, 40.9 ± 2.14, 38.7 ± 2.83, and 0.6 ± 0.03 U/mg protein, respectively, which increased linearly (*P* < 0.05) and quadratically (*P* < 0.05) and were close to the normal control group. These results were consistent with the free radical scavenging results and suggested that LW-XG had a protective effect on Caco-2 cells injured by H_2_O_2_.

## 4. Conclusions

Due to the need for polysaccharides with a reduced or confined molecular weight within a narrow range in different applications, the degradation of xanthan gum has attracted much attention in recent years. In the present work, the endophytic fungus *C. globosum* CGMCC 6882 was used to biodegrade commercial xanthan and a low-molecular-weight xanthan gum (4.07 × 10^4^ Da) was successfully obtained. LW-XG exhibited good free-radical scavenging activity and excellent protective effect on Caco-2 cells injured by H_2_O_2_. In future work, xanthan oligosaccharides with different molecular weights will not only be efficiently produced by controlling the biodegradation conditions and using genetic engineering methods, but the bioactivities of different xanthan oligosaccharides will be studied to examine the structure–activity relationships of polysaccharides.

## Figures and Tables

**Table 1:** Effect of LW-XG on SOD, CAT, GSH-Px, and MDA activity. Different letters represent a significant difference at *P* < 0.05.

Parameters (U/mg protein)	Normal Control Group	Model Control Group	LW-XG Concentration (mg/mL)		
			0.75	1.50	3.00
SOD	52.1 ± 1.95 ^a^	21.8 ± 2.06 ^b^	29.4 ± 1.79 ^c^	39.8 ± 3.15 ^c^	51.4 ± 2.63 ^c^
CAT	43.5 ± 2.17 ^a^	22.9 ± 2.89 ^b^	27.2 ± 2.75 ^c^	35.9 ± 1.97 ^c^	40.9 ± 2.14 ^c^
GSH-Px	41.1 ± 3.13 ^a^	20.1 ± 2.57 ^b^	25.0 ± 2.91 ^c^	33. 7 ± 2.10 ^c^	38.8 ± 2.83 ^c^
MDA	0.5 ± 0.08 ^a^	1.6 ± 0.15 ^b^	1.3 ± 0.10 ^c^	0.8 ± 0.07 ^c^	0.6 ± 0.03 ^c^
